# Identification of 14-3-3 Proteins Phosphopeptide-Binding Specificity Using an Affinity-Based Computational Approach

**DOI:** 10.1371/journal.pone.0147467

**Published:** 2016-02-01

**Authors:** Zhao Li, Jijun Tang, Fei Guo

**Affiliations:** 1 School of Computer Science and Technology, Tianjin University, 92 Weijin Road, Nankai District, Tianjin, P.R. China; 2 School of Computational Science and Engineering, University of South Carolina, Columbia, United States of America; Russian Academy of Sciences, Institute for Biological Instrumentation, RUSSIAN FEDERATION

## Abstract

The 14-3-3 proteins are a highly conserved family of homodimeric and heterodimeric molecules, expressed in all eukaryotic cells. In human cells, this family consists of seven distinct but highly homologous 14-3-3 isoforms. 14-3-3*σ* is the only isoform directly linked to cancer in epithelial cells, which is regulated by major tumor suppressor genes. For each 14-3-3 isoform, we have 1,000 peptide motifs with experimental binding affinity values. In this paper, we present a novel method for identifying peptide motifs binding to 14-3-3*σ* isoform. First, we propose a sampling criteria to build a predictor for each new peptide sequence. Then, we select nine physicochemical properties of amino acids to describe each peptide motif. We also use auto-cross covariance to extract correlative properties of amino acids in any two positions. Finally, we consider elastic net to predict affinity values of peptide motifs, based on ridge regression and least absolute shrinkage and selection operator (LASSO). Our method tests on the 1,000 known peptide motifs binding to seven 14-3-3 isoforms. On the 14-3-3*σ* isoform, our method has overall pearson-product-moment correlation coefficient (PCC) and root mean squared error (RMSE) values of 0.84 and 252.31 for *N*–terminal sublibrary, and 0.77 and 269.13 for *C*–terminal sublibrary. We predict affinity values of 16,000 peptide sequences and relative binding ability across six permutated positions similar with experimental values. We identify phosphopeptides that preferentially bind to 14-3-3*σ* over other isoforms. Several positions on peptide motifs are in the same amino acid category with experimental substrate specificity of phosphopeptides binding to 14-3-3*σ*. Our method is fast and reliable and is a general computational method that can be used in peptide-protein binding identification in proteomics research.

## Introduction

The 14-3-3 proteins are a highly conserved family of homodimeric and heterodimeric molecules, expressed in all eukaryotic cells [1]. As a key regulator of signal transduction, 14-3-3 isoforms participate in important cellular events including regulation of apoptosis, adhesion-dependent integrin signaling, cell cycle control, DNA damage, metabolism and transcriptional regulation [2]. We have been particularly interested in understanding roles of different 14-3-3 isoforms in cell proliferation, cell cycle control, and human tumorigenesis.

In human cells, this family of proteins consists of seven distinct but highly homologous 14-3-3 isoforms: *β*, *ϵ*, *η*, *γ*, *σ*, *τ*, *ζ* [3]. Phosphate can bind to all of the 14-3-3 family and therefore being present at high intracellular concentration [4, 5]. With roles of different 14-3-3 isoforms in a wide variety of signal transduction processes, 14-3-3*σ* is the only isoform directly linked to cancer in epithelial cells, which is regulated by major tumor suppressor genes [6–8]. The stabilizing ring-ring and salt bridge interactions unique to the 14-3-3*σ* homodimer structure are revealed by the x-ray crystal structure of 14-3-3*σ* with binding peptide, which potentially destabilized electrostatic interactions between subunits in 14-3-3*σ*-containing heterodimers, and rationalized preferential homodimerization of 14-3-3*σ* in vivo. The interaction of the phosphopeptide with 14-3-3 reveals a conserved mechanism for phospho-dependent ligand binding, implying that the phosphopeptide binding cleft is not the critical determinant of the unique biological properties of 14-3-3*σ*.

There exist many approaches identify substrate specificity of phosphopeptides that preferentially bind to 14-3-3*σ* over other isoforms. A major advance in understanding 14-3-3 phosphopeptide binding specificity was the recognition by Yaffe et al. [4] Using phosphoserine-oriented peptide libraries, they identified a consensus hexapeptide binding motif, *RXXpSXP*, binding to all known 14-3-3 isoforms. The basic residue *X* means any of 20 amino acid types. Erik et al. [9] solved the x-ray crystal structure of 14-3-3*σ*, which provided structure information and demonstrated that 14-3-3*σ* preferentially form homodimers in cell. Unlike other six isoforms, they identified a second ligand binding sites involved in 14-3-3*σ*-specific ligand discrimination. In order to identify phosphopeptides that preferentially bind to 14-3-3*σ* over other isoforms, Lu et al. [10] used fragment-based combinatorial peptide microarray platform, dividing whole library into *N*–terminal and *C*–terminal sublibraries *P*_−3_ *P*_−2_ *P*_−1_ − *p*(*S*/*T*) − *P*_+1_ *P*_+2_ *P*_+3_. The (+/−) represents relative position of *p*(*S*/*T*), and *P*_+/−_ represents ten or five individual amino acids in each position. Ten different amino acid building blocks (*R*, *E*, *F*, *L*, *Q*, *A*, *G*, *V*, *K*, *P*) for *P*_+/−1_ *P*_+/−2_ and a total of five different amino acid building blocks (*R*, *E*, *F*, *L*, *P*) for *P*_+/−3_ positions were used. The phosphopeptide library was synthesized to get 14-3-3*σ*-specific binding peptide. They confirmed the previous consensus binding motif by Yaffe, and finally identified two 14-3-3*σ*-specific binders. However, their experimental methods are expensive and time consuming. Sequence variation at other positions near the phosphorylated site can cause differences in binding affinities, thus we can use the physical-chemical information to construct a computational model to extrapolate 14-3-3*σ*-specific binders from experimental data.

Roughly speaking, three categories of computational methods for detecting protein interactions exist. They are based on the evolution of information, natural language processing, the feature of the amino acid sequence and three-dimensional structural information. First, the evolution information [11] is extracted from multiple sequence alignment of homologous proteins. Family tree similarities are quantify tree similarities implemented a simple linear correlation between distance matrices of two protein families, as a proxy of their phylogenetic trees [12–15]. However, their computational tasks are huge. Second, methods based on Natural Language Processing (NLP) [16] can find the evidence for protein interactions from relevant scientific literatures. The problem is some binding information can not entirely appear in the literature in time. Using the hidden internal structure buried into noisy amino acid sequences [17–19] and some machine learning algorithms, some researchers propose prediction methods only using protein sequence information. Using three-dimensional structural information, Zhang et al. [20] predicted protein interaction with a considerable accuracy and coverage that are superior to predictions based non-structural evidence. Base on pairwise similarity method and primary structure of protein, Zaki et al. [21] measured similarity between protein sequences to predict protein binding residues. Since 14-3-3 phosphopeptide binders only have six meaningful positions in binding motif sequences, the state-of-the-art methods must be not suitable for this issue, how to dig the useful and important features is the first challenge.

In this paper, we propose the first computational method to identify and analysis 14-3-3 phosphopeptide binding specificity. We present a novel method for identifying peptide motifs binding to 14-3-3 isoforms. First, we propose a sampling criteria to build a predictor for each new peptide motif. Then, we select nine physicochemical properties of amino acids to describe each peptide motif. We also use auto cross covariance [22, 23] to extract correlative properties of amino acids in any two positions. Finally, we consider elastic net [24] to predict affinity values of peptide motifs, based on ridge regression and least absolute shrinkage and selection operator (LASSO). Our method verifies 1,000 known peptide motifs binding to seven distinct but highly homologous 14-3-3 isoforms. On 14-3-3*σ* isoform, our method has overall pearson-product-moment correlation coefficient (PCC) and root mean squared error (RMSE) values of 0.84 and 252.31 for *N*–terminal sublibrary, and 0.77 and 269.13 for *C*–terminal sublibrary. It demonstrates the rationality of our computational method. Our method tests on 16,000 peptide sequences to predict binding affinity values, and relative binding ability across six permutated positions similar with the experimental value. We identify phosphopeptides that preferentially bind to 14-3-3*σ* over other isoforms. Several positions on peptide motifs are in the same amino acid category with experimental substrate specificity of phosphopeptides binding to 14-3-3*σ*.

## Materials and Methods

**Fig 1:**
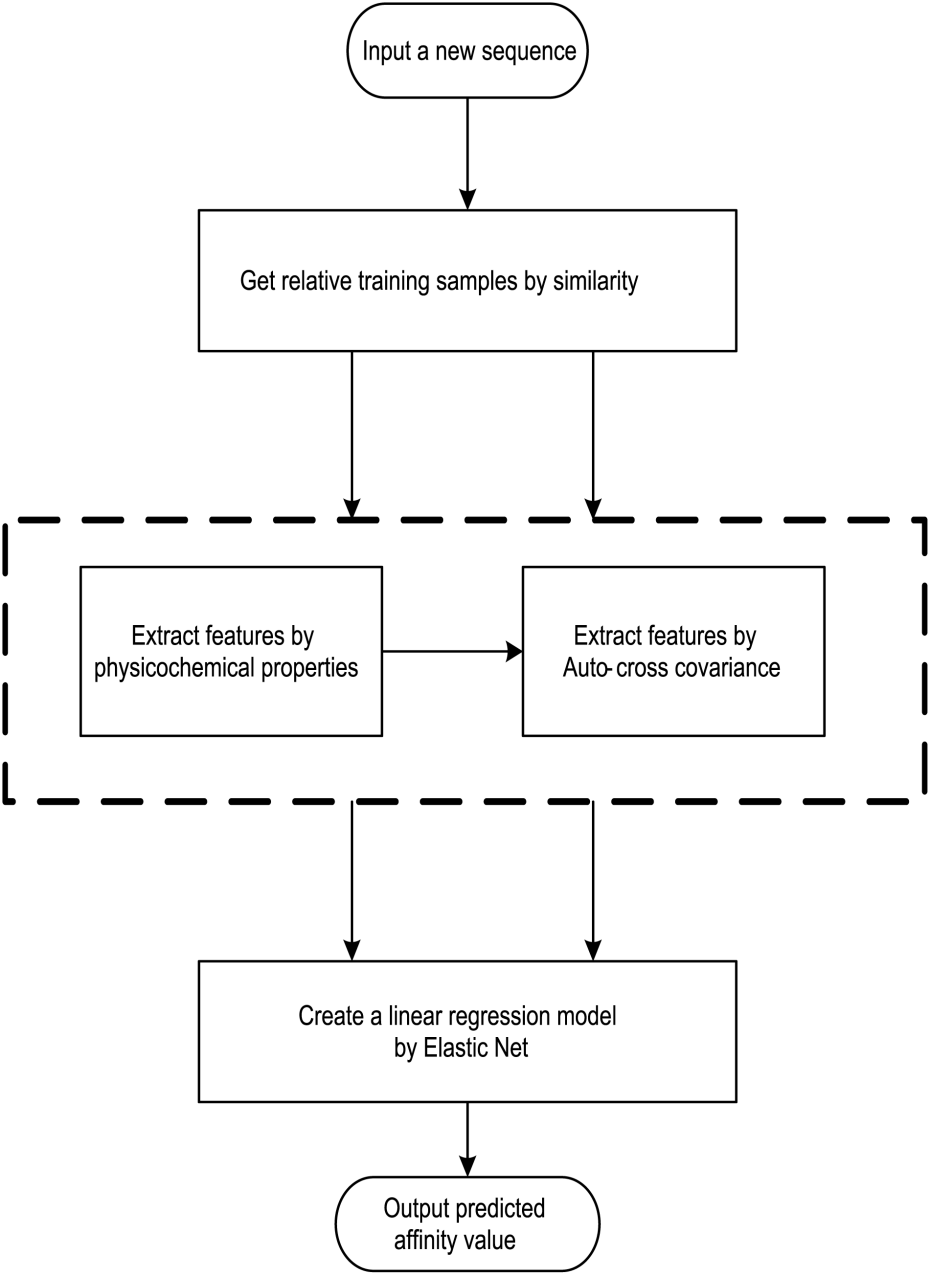
The architecture of the computational approach to identifying 14-3-3 Proteins Phosphopeptide-Binding Specificity.

We present an affinity-based computational approach for identifying peptide motifs binding to 14-3-3 isoforms, and this novel method is also the first computational method of 14-3-3 proteins phosphopeptide-binding specificity identification. For each 14-3-3 isoform, we have 1,000 peptide motifs with experimental binding affinity values, treated as known in this study. We need to identify affinity values of 16,000 peptide sequences binding to seven 14-3-3 isoforms. First, we propose a sampling criteria to build a predictor for each new peptide motif. Then, we select nine physicochemical properties of amino acids to describe each peptide motif. We also use auto cross covariance to extract correlative properties of amino acids in any two positions. Finally, we consider elastic net to predict affinity values of peptide motifs, based on ridge regression and least absolute shrinkage and selection operator (LASSO). The method flow is shown in Fig 1.

### Data Set

Lu [10] proposed a fragment-based combinatorial peptide microarray, which enables sufficient coverage of all (*P*_−3_ *P*_−2_ *P*_−1_ − *p*(*S*/*T*) − *P*_+1_ *P*_+2_ *P*_+3_) sequences with only 1,000 peptide motifs (500 *N*–terminal and *C*–terminal sublibraries). These peptide motifs are formed as a phosphopeptide library. In a predefined manner, they use a total of ten different amino acid building blocks (*R*, *E*, *F*, *L*, *Q*, *A*, *G*, *V*, *K*, *P*) for *P*_+/−1_ and *P*_+/−2_ positions, and a total of five different amino acid building blocks (*R*, *E*, *F*, *L*, *P*) for *P*_+/−3_ position.

With respect to each *N*–terminal and *C*–terminal, there are 5 × 10 × 10 possibilities. For each 14-3-3 isoform, we have 1,000 peptide motifs with experimental binding affinity values. In order to study 14-3-3 proteins phosphopeptide-binding specificity from a global search space, which means there are 20 × 20 × 20 possibilities in each *N*–terminal and *C*–terminal. We will identify affinity values of 16,000 peptide sequences binding for seven 14-3-3 isoforms. To maximize the number of peptide motifs, twenty amino acids, instead of ten and five, are used at *P*_+/−1_, *P*_+/−2_ and *P*_+/−3_ positions.

### Sampling Criteria

We propose a sampling criteria to build a predictor for each new peptide motif. If all 500 peptide motifs for one terminal are used to construct a regression model, the predictor would be confused due to importing many irrelevant peptide sequences. For each new peptide sequence, we only select relevant peptide motifs to construct a dynamic regression model, which can improve average precision of the predictor.

All amino acids can be divided into five categories [25]: amino acids with positive charged side chains, amino acids with negative charged side chains, amino acids with polar uncharged side chains, amino acids with hydrophobic side chains and special cases. The details are shown in Table 1. For each new peptide sequence, we select the relevant peptide motifs with at least one *P*_1/2/3_ position in the same category.

**Table 1:** Five categories of 20 amino acids.

Category	Amino Acids ^a^
Amino Acids with Positive Charged Side Chains	R, H, K
Amino Acids with Negative Charged Side Chains	D, E
Amino Acids with Polar Uncharged Side Chains	S, T, N, Q
Amino Acids with Hydrophobic Side Chains	A, I, L, M, F, W, Y, V
Special Cases	C, G, P

^a^ Standard abbreviations are used for all amino acids.

### Feature Extraction

Based on relevant peptide motifs, we extract a set of features from the peptide sequences. There are two kinds of features in this study: one extracts nine physicochemical properties for each position and this produces 27 features; the other extracts correlation of amino acids in any two positions by auto-cross covariance, nine features for every two positions, thus leads to another 27 features [26].

We select nine physicochemical properties of all 20 amino acid types to describe each peptide motif: hydrophobicity, hydrophicility, volumes of side chains, polarity, polarizability, solvent-accessible surface area (SASA), net charge index (NCI) of side chains, mass, and hydrogen bond. Details are shown in Table 2 [26]. These nine physicochemical properties are normalized to zero mean and unit standard deviation [22, 26], and the first kind of 27 features can be extracted by these normalized properties as follows:

$$\begin{array}{c} P_{i,j}^{\prime }=\frac{P_{i,j}-P_{j}}{S_{j}} \end{array}$$ (1)

where *P*_*j*_ represents the mean of the *j*-th property, *P*_*i*,*j*_ is the *j*-th property of the *i*-th amino acid, *S*_*j*_ is the corresponding unit standard deviation.

**Table 2:** Nine physicochemical properties for 20 amino acid types.

	Physicochemical Properties ^a^								
	*H*_1_	*H*_2_	*H*_3_	*V*	*P*_1_	*P*_2_	SASA	NCI	MASS
A	0.62	-0.5	2	27.5	8.1	0.046	1.181	0.007187	71.0788
C	0.29	-1	2	44.6	5.5	0.128	1.461	-0.03661	103.1388
D	-0.9	3	4	40	13	0.105	1.587	-0.02382	115.0886
E	-0.74	3	4	62	12.3	0.151	1.862	0.006802	129.1155
F	1.19	-2.5	2	115.5	5.2	0.29	2.228	0.037552	147.1766
G	0.48	0	2	0	9	0	0.881	0.179052	57.0519
H	-0.4	-0.5	4	79	10.4	0.23	2.025	-0.01069	137.1411
I	1.38	-1.8	2	93.5	5.2	0.186	1.81	0.021631	113.1594
K	-1.5	3	2	100	11.3	0.219	2.258	0.017708	128.1741
L	1.06	-1.8	2	93.5	4.9	0.186	1.931	0.051672	113.1594
M	0.64	-1.3	2	94.1	5.7	0.221	2.034	0.002683	131.1986
N	-0.78	2	4	58.7	11.6	0.134	1.655	0.005392	114.1039
P	0.12	0	2	41.9	8	0.131	1.468	0.239531	97.1167
Q	-0.85	0.2	4	80.7	10.5	0.18	1.932	0.049211	128.1307
R	-2.53	3	4	105	10.5	0.18	1.932	0.049211	156.1875
S	-0.18	0.3	4	29.3	9.2	0.062	1.298	0.004627	87.0782
T	-0.05	-0.4	4	51.3	8.6	0.108	1.525	0.003352	101.1051
V	1.08	-1.5	2	71.5	5.9	0.14	1.645	0.057004	99.1326
W	0.81	-3.4	3	145.5	5.4	0.409	2.663	0.037977	186.2132
Y	0.26	-2.3	3	117.3	6.2	0.298	2.368	0.023599	163.1760

^a^ *H*_1_, hydrophobicity; *H*_2_, hydrophicility; *H*_3_, hydrogen bond; *V*, volumes of side chains; *P*_1_, polarity; *P*_2_, polarizability; SASA, solvent-accessible surface area; NCI, net charge index of side chains; MASS, average mass of amino acid.

We also use auto-cross covariance to extract correlation of amino acids in any two positions. Auto-cross covariance (ACC) can get two kinds of variables, auto cross (AC) between the same descriptor, and cross covariance (CC) between two different descriptors. In this study, we only use AC variables in order to avoid generating too large number of variants. We modify the AC variables to get correlation of amino acids in any two positions as follows:

$$\begin{array}{c} AC_{\left(m,n,j\right)}=\left(X_{m,j}-\frac{1}{3}\sum_{i=1}^{3}X_{i,j}\right)\times \left(X_{n,j}-\frac{1}{3}\sum_{i=1}^{3}X_{i,j}\right) \end{array}$$ (2)

where *m*, *n* are different position of a peptide and *j* is the *j*-th property of residues, *X*_*i*,*j*_ is the *j*-th property of residue on the *i*-th position.

### Linear Regression

After feature extraction described above, a suitable regression model should be selected to built an accurate predictor. Linear regression is one of the most widely used regression model in mathematical statistics, which has very good interpretability [27]. It not only gets a series of regression coefficient, but also explains how important one variable is, thus is very important in this study. We consider naive linear regression model to built an accurate predictor. Given feature vectors *X*_1_, ⋯, *X*_*p*_ describing *p* features on each peptide sequence, we identify its corresponding value *f*(*X*) to represent binding affinity value as follows:

$$\begin{array}{c} f\left(X\right)=\beta_{0}+\sum_{j=1}^{p}X_{j}\beta_{j} \end{array}$$ (3)

Different linear regression models, i.e. ridge regression and LASSO, adopt different methods to minimize the residual sum of squares (RSS). Ridge regression minimizes the RSS subject to a bound on L2-norm of coefficients as follows:

$$\begin{array}{c} arg\;\underset{\beta }{\text{min}}\{\sum_{i=1}^{N}{\left(y_{i}-\beta_{0}-\sum_{j=1}^{p}x_{ij}\beta_{j}\right)}^{2}+\lambda \sum_{j=1}^{p}\beta_{j}^{2}\} \end{array}$$ (4)

where *λ* controls the penalty of coefficient size, and *N* is the number of peptide motifs.

LASSO tends to truncate some coefficients exactly at zero and hence makes model interpretable [28, 29]. It minimizes RSS subject to a bound on L1-norm of coefficients [28], which is the sum of absolute values of coefficients, the equation is as follows:

$$\begin{array}{c} arg\;\underset{\beta }{\text{min}}\{\sum_{i=1}^{N}{\left(y_{i}-\beta_{0}-\sum_{j=1}^{p}x_{ij}\beta_{j}\right)}^{2}+\lambda \sum_{j=1}^{p}|\beta_{j}|\} \end{array}$$ (5)

Considering pairwise correlations between 54 variables, we use elastic net to predict affinity values of peptide motifs. Zou [24, 30] proposed elastic net, a new regularization and variable selection method, which combines ridge regression and LASSO by making a trade-off in these two penalties. The elastic net calculates corresponding value of each peptide sequence as follows:

$$\begin{array}{c} arg\;\underset{\beta }{\text{min}}\{\sum_{i=1}^{N}{\left(y_{i}-\beta_{0}-\sum_{j=1}^{p}x_{ij}\beta_{j}\right)}^{2}+\lambda P_{\alpha }\left(\beta \right)\} \end{array}$$ (6)

where

$$\begin{array}{c} P_{\alpha }\left(\beta \right)=\sum_{j=1}^{p}\left[\frac{1}{2}\left(1-\alpha \right)\beta_{j}^{2}+\alpha |\beta_{j}|\right] \end{array}$$ (7)

We can calculate a ten-fold cross-validation to get the optimal *λ* for elastic net. In order to find the most suitable *α*, we produce a sequence from 0 to 1 with interval of 0.1. We apply 11 values of *α* to get the most suitable predictor.

## Results

In this section, we have done three kinds of experiments. First, our method verifies the 1,000 known peptide motifs binding to seven distinct but highly homologous 14-3-3 isoforms. Second, our method tests on 16,000 peptide sequences to predict binding affinity values. Third, we identify phosphopeptides that preferentially bind to 14-3-3*σ* over other isoforms.

### Verification on 1,000 known peptide motifs

Our method verifies 1,000 peptide motifs binding to seven 14-3-3 isoforms. The Pearson-product-moment correlation coefficient (PCC) and the root mean squared error (RMSE) [31] are used to evaluate performance as follows:

$$\begin{array}{c} PCC=\sqrt{1-\frac{\sum_{i=1}^{N}{\left(e_{i}-p_{i}\right)}^{2}}{\sum_{i=1}^{N}{\left(e_{i}-\bar{e}\right)}^{2}}} \end{array}$$ (8)

and

$$\begin{array}{c} RMSE=\sqrt{\frac{\sum_{i=1}^{N}{\left(e_{i}-p_{i}\right)}^{2}}{\left|D\right|}} \end{array}$$ (9)

where *D* contains all of relevant binding motifs, $\bar{e}$ is the average binding affinity, *e*_*i*_ denotes experimental binding affinity value of the *i*-th peptide sequence, *p*_*i*_ denotes the predicted affinity value of the *i*-th peptide sequence. An accurate predictor will get *PCC* = 1, *RMSE* = 0.

We using the 999 peptide motifs with experimental binding affinity values as training data, removing the predicted peptide sequence. When only selecting ‘relevant’ data for building the predictor, about 300 peptide motifs are selected as training data each time on average. Details on identifying peptide motifs binding to 14-3-3 isoforms are shown in Table 3. On the 14-3-3*σ* isoform, our method has overall PCC and RMSE values of 0.84 and 252.31 for *N*–terminal sublibrary, and 0.77 and 269.13 for *C*–terminal sublibrary. It yields a considerable PCC in all seven isoforms, and the results clearly highlight the effectiveness of our method. At the same time, the RMSE values vary in different isoforms, because of several extra large values of affinity and imbalance peptide distribution between diverse values in different isoforms.

**Table 3:** Details on predicting peptide motifs binding to 14-3-3 isoforms.

	N-terminal		C-terminal	
	PCC	RMSE	PCC	RMSE
*σ*	0.84	252.31	0.77	269.13
*β*	0.72	229.12	0.63	245.10
*ϵ*	0.83	417.38	0.75	491.73
*η*	0.81	230.83	0.71	252.94
*γ*	0.86	470.08	0.79	463.40
*τ*	0.78	637.67	0.72	678.95
*ζ*	0.87	2087.20	0.81	2365.42

For each peptide motif to be predicted, we use ten-folds cross-validation to get the most appropriate regression model. The cross-validation results over 1,000 peptides are as showed in S1 Table.

#### Comparison to Experimental Techniques

**Fig 2:**
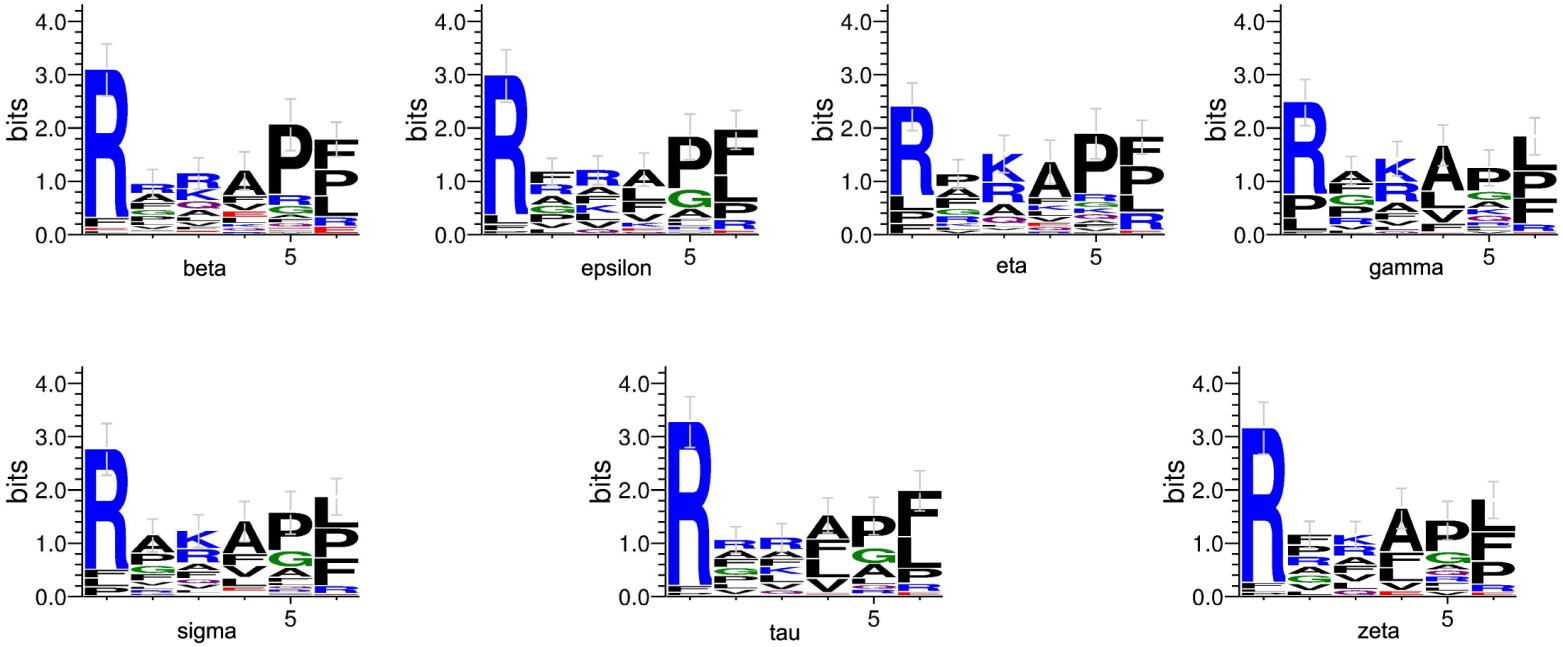
Position-specific scoring matrix on top 50 motifs identified from 1,000 peptide sequences against individual 14-3-3 isoforms.

**Fig 3:**
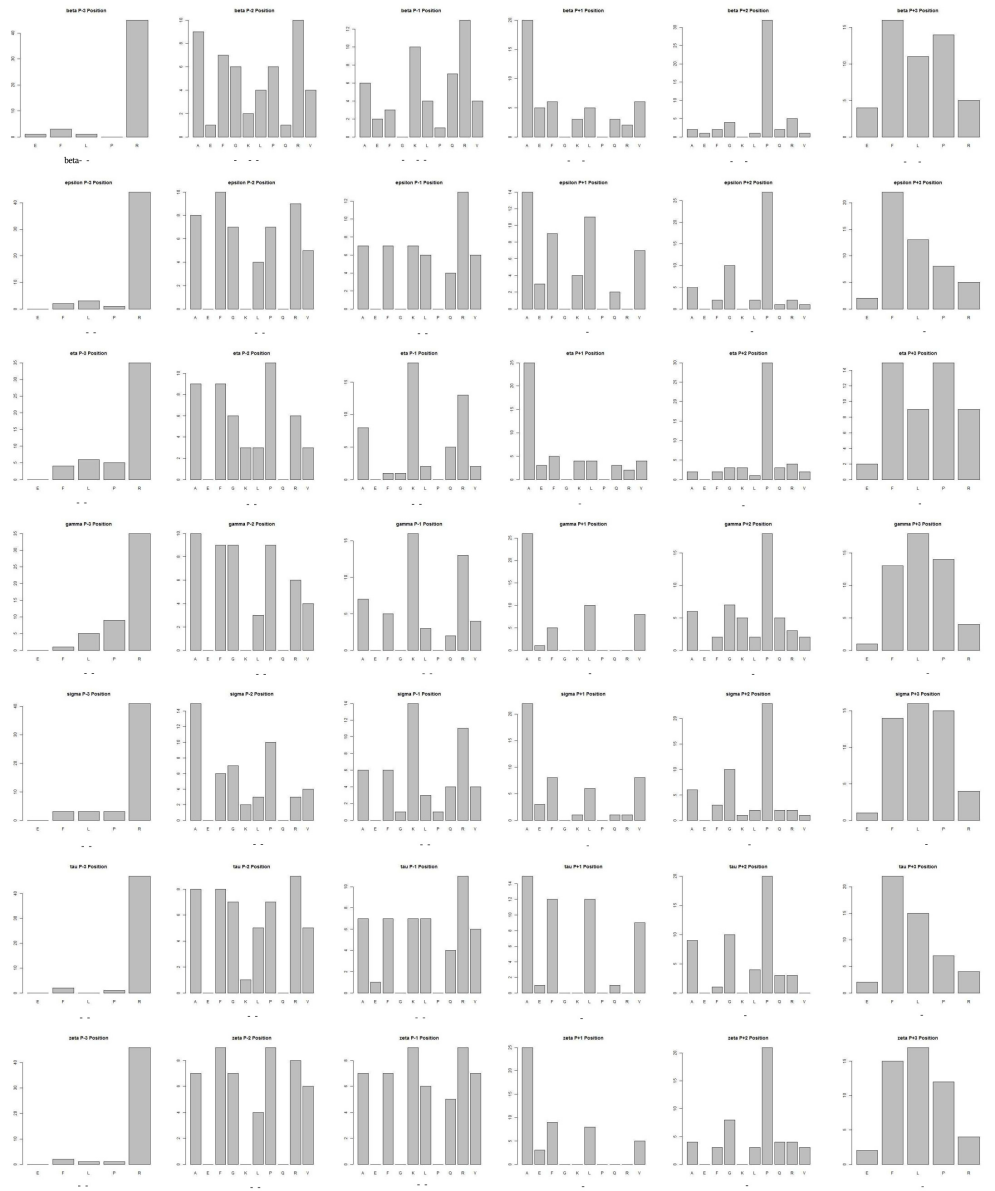
Binding affinity of seven 14-3-3 isoforms across six positions from top-50 peptides from both N- and C-terminal sublibrary.

We produce a position-specific scoring matrix [32] on the top 50 motifs identified from each *N*–terminal and *C*–terminal sublibrary against each individual 14-3-3 isoform, to reflect position specialty for each amino acid, as shown in Fig 2. The height of each letter represents weighted contribution of that amino acid to the overall peptide binding. Our method is compared with the experimental methods from Lu [10], as summarized in Table 4. Our computational results are consistent with the previous experimental works on 14-3-3 isoforms binding peptide motifs. We get relative binding ability of all seven 14-3-3 isoforms across six permutated positions, as shown in Fig 3. Each bar represents the frequency of a particular amino acid. This confirms highly homologous feature of 14-3-3 isoforms, similar with consensus binding motif *RXXpSXP*. It is obvious that all of the seven isoforms strongly select peptide motifs containing Arg on *P*_−3_ position and Pro on *P*_+2_ position.

**Table 4:** 14-3-3 preferences determined with different methods on 1,000 peptide motifs.

	Position Relative to p(S/T)					
	*P*_−3_	*P*_−2_	*P*_−1_	*P*_+1_	*P*_+2_	*P*_+3_
H.S. Lu	**R**	**PFR**A	**RK**	**AVFL**	**P**A	**FPL**
Our Method	**R**KPF	**PFR**G	**RK**F	**AVFL**	**P**GR	**FPL**R

#### Comparison to Computational Methods

In this study, we use Elastic Net as regression model, which gets a better result and costs less time, comparing to other techniques. The quantitative comparison with other techniques, such as Simple Linear Regression, Support Vector Regression with RBF kernel and Neural Network with one hidden layer, are as show in Table 5.

**Table 5:** Prediction results of peptide motifs binding to 14-3-3 isoforms by different regression techniques.

	Elastic Net		Simple Linear Regression		Support Vector Regression		Neural Network	
	PCC	RMSE	PCC	RMSE	PCC	RMSE	PCC	RMSE
N-terminal								
*σ*	0.84	252.31	0.82	261.69	0.79	283.16	0.60	368.39
*β*	0.72	229.12	0.69	238.40	0.70	236.18	0.57	270.43
*ϵ*	0.83	417.38	0.82	498.71	0.80	529.34	0.64	675.74
*η*	0.81	230.83	0.80	238.09	0.79	239.43	0.55	327.70
*γ*	0.86	470.08	0.86	474.16	0.83	506.56	0.59	745.79
*τ*	0.78	637.67	0.78	637.58	0.75	669.53	0.56	844.41
*ζ*	0.87	2087.20	0.88	2042.67	0.84	2306.04	0.56	3526.35
C-terminal								
*σ*	0.77	269.13	0.76	273.19	0.74	279.54	0.64	321.78
*β*	0.63	245.10	0.61	247.96	0.59	252.64	0.51	269.64
*ϵ*	0.75	491.73	0.74	479.30	0.73	483.90	0.63	550.81
*η*	0.71	252.94	0.69	256.66	0.69	257.90	0.48	311.73
*γ*	0.79	463.40	0.79	459.40	0.80	454.01	0.68	558.68
*τ*	0.72	678.95	0.71	686.52	0.70	691.33	0.59	786.58
*ζ*	0.81	2365.42	0.80	2352.32	0.79	2429.84	0.66	3012.30

On the 14-3-3*σ* isoform, Elastic Net has overall PCC and RMSE values of 0.84 and 252.31 for *N*–terminal sublibrary, and 0.77 and 269.13 for *C*–terminal sublibrary. However, Simple Linear Regression has overall PCC and RMSE values of 0.82 and 261.69 for *N*–terminal sublibrary, and 0.76 and 273.19 for *C*–terminal sublibrary; Support Vector Regression with RBF kernel has overall PCC and RMSE values of 0.79 and 283.16 for *N*–terminal sublibrary, and 0.74 and 279.54 for *C*–terminal sublibrary; Neural Network with one hidden layer has overall PCC and RMSE values of 0.60 and 368.39 for *N*–terminal sublibrary, and 0.64 and 321.78 for *C*–terminal sublibrary. For seven 14-3-3 isoforms, our method using Elastic Net can outperform other excellent regression techniques.

### Prediction on 16,000 peptide sequences

**Fig 4:**
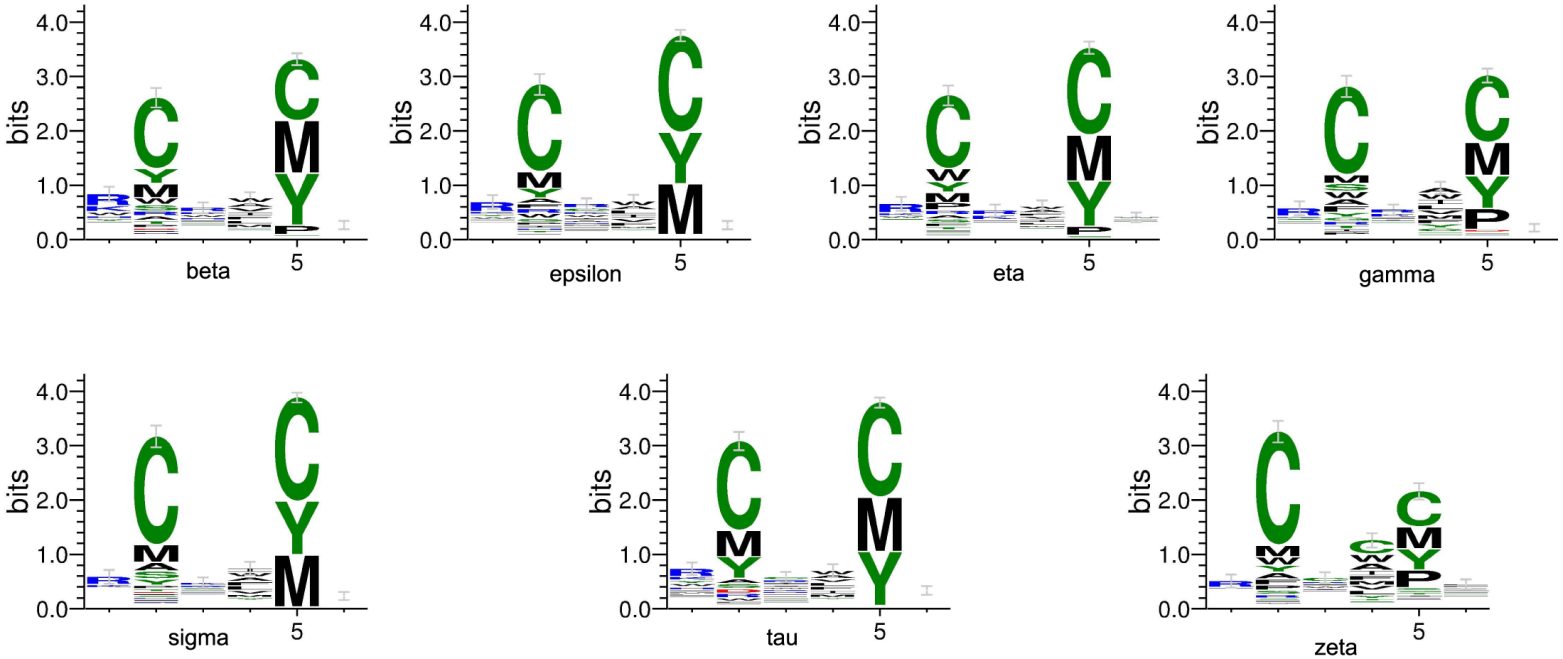
Position-specific scoring matrix on top 500 motifs identified from 16,000 peptide sequences against individual 14-3-3 isoforms.

**Fig 5:**
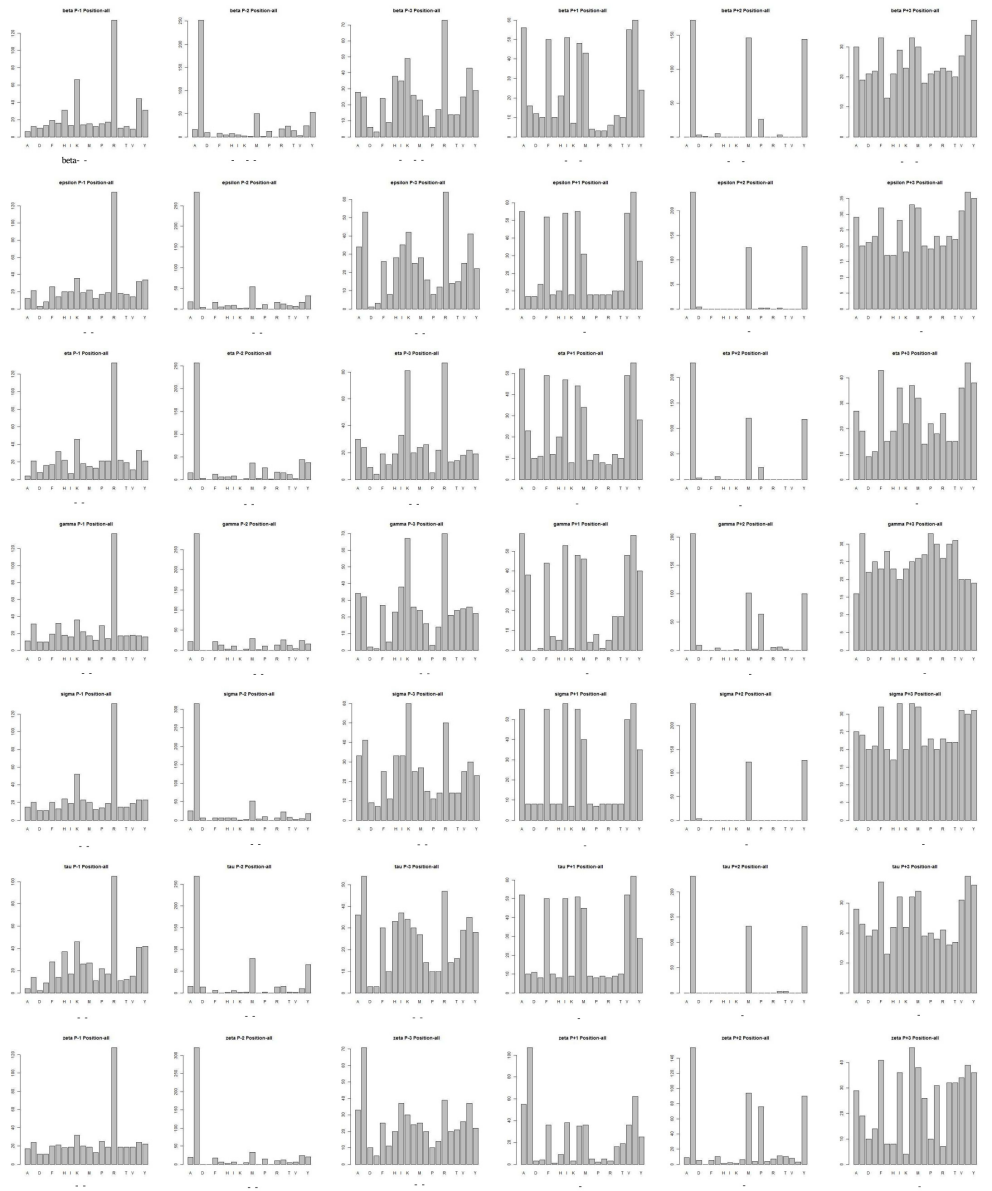
Binding affinity of seven 14-3-3 isoforms across six positions from top-500 peptides from both N- and C-terminal sublibrary.

We using the 1,000 peptide motifs with experimental binding affinity values as training data, and aim to predict affinity values of 16,000 motifs for each 14-3-3 isoform. Our method predicts affinity values of all 16,000 peptide sequences binding to seven 14-3-3 isoforms. Our results confirm highly conserved binding specificity amongst 14-3-3 isoforms, and uncover some new binding information. We produce a position-specific scoring matrix on the top 500 motifs identified from each *N*–terminal and *C*–terminal sublibrary against individual 14-3-3 isoforms, to reflect position specialty for each amino acid, as shown in Fig 4. We get the relative binding ability of seven 14-3-3 isoforms across six permutated positions, as shown in Fig 5.

Our method is compared with the experimental methods from Yaffe [4], as summarized in Table 6. We find the relative binding ability across six permutated positions, which are similar with the experimental results. All of the seven isoforms select peptide motifs containing Arg or Lys on *P*_−3_ position; Cys and amino acids with hydrophobic side chain on *P*_−2_ position; basic residues on *P*_−1_ and *P*_+3_ positions, and amino acids with hydrophobic side chain having most of aromatic residues on *P*_+1_ position. On *P*_+2_ position, peptide motifs with Cys, Tyr, Met and Pro show strong selection; however there is just Pro in Yaffe’s research, it may be because that Yaffe used all amino acids except Cys.

**Table 6:** 14-3-3 preferences determined with different methods on 16,000 peptide sequences.

	Position Relative to p(S/T)					
	*P*_−3_	*P*_−2_	*P*_−1_	*P*_+1_	*P*_+2_	*P*_+3_
Yaffe	**RK**	**YASW**FH	RKH	**WAFL**Y	**P**G	**X**
Our Method	**RK**	**YASW**CM	X	**WAFL**IVM	**P**CMY	**X**

### Specificity of 14-3-3*σ* binding peptide motifs

On the 1,000 known peptide motifs, we identify the top 100 peptide motifs, irrespective of *N*–terminal or *C*–terminal, binding each 14-3-3 isoform. We filter and identify consensus sequences present in all seven isoforms, giving a total of 51 unique peptide motifs, as shown in Table 7. Compared with Lu [10], 30 peptide motifs of our results are the same with experimental 46 binding sequences, which are represented by the ⋆ label. In the same time, most of the left 21 peptides have the same type of amino acids in two positions. The precision and recall values for our method are 59% and 65%, respectively. It indicates that our computational method obtains great consistence with experiment results.

**Table 7:** List of 51 consensus top binders from 1,000 peptide sequences against all seven 14-3-3 isoforms.

No.		N-terminal	No.		N-terminal	No.		C-terminal
1		FFRpS/TXXX^b^	20		RLRpS/TXXX	36		XXXpS/TAGF
2		RAApS/TXXX	21	*	RPApS/TXXX	37		XXXpS/TAGP
3	*^a^	RAFpS/TXXX	22	*	RPKpS/TXXX	38	*	XXXpS/TAPF
4	*	RAKpS/TXXX	23	*	RPLpS/TXXX	39	*	XXXpS/TAPL
5	*	RALpS/TXXX	24	*	RPQpS/TXXX	40	*	XXXpS/TAPP
6	*	RAQpS/TXXX	25	*	RPRpS/TXXX	41		XXXpS/TAPR
7	*	RARpS/TXXX	26		RPVpS/TXXX	42	*	XXXpS/TFPF
8	*	RAVpS/TXXX	27		RRApS/TXXX	43	*	XXXpS/TFPL
9	*	RFApS/TXXX	28	*	RRFpS/TXXX	44		XXXpS/TFPP
10	*	RFFpS/TXXX	29	*	RRKpS/TXXX	45		XXXpS/TLPF
11	*	RFKpS/TXXX	30		RRLpS/TXXX	46	*	XXXpS/TLPL
12	*	RFRpS/TXXX	31	*	RRQpS/TXXX	47		XXXpS/TLPP
13		RGApS/TXXX	32		RRRpS/TXXX	48		XXXpS/TLPR
14		RGKpS/TXXX	33	*	RVApS/TXXX	49	*	XXXpS/TVPF
15		RGQpS/TXXX	34	*	RVKpS/TXXX	50	*	XXXpS/TVPL
16		RGRpS/TXXX	35	*	RVRpS/TXXX	51	*	XXXpS/TVPP
17		RGVpS/TXXX						
18		RLApS/TXXX						
19		RLKpS/TXXX						

^a^ The motif with label * is the same with experimental binding sequences of H.S. Lu.

^b^ The basic residue *X* means any of 20 amino acid types.

We identify four peptide motifs that have 14-3-3*σ* specificity, as shown in Table 8. The four peptide motifs belong to the top 100 sequences binding 14-3-3*σ*, but not being part of the top 100 sequences binding other 14-3-3 isoforms. Compared with two 14-3-3*σ* preferable binders of Lu, B1:LFGpSLLR and B2:LFGpSLVR, three motifs have residues in the same amino acid category on *P*_−2_ and *P*_+1_ positions, as shown in Table 1. On *P*_−2_ position, Ala along with Phe has Hydrophobic side chain; Phe and Leu on *P*_+1_ position have polar uncharged side chains simultaneously.

**Table 8:** List of four preferable binders of 14-3-3*σ* from 1,000 peptide sequences.

No.	N-terminal	No.	C-terminal
1	RAGpS/TXXX	4	XXXpS/TFGP
2	EAKpS/TXXX		
3	RGGpS/TXXX		

We define a similarity score between the our predicted 14-3-3*σ*-specific motifs and Lu’s findings. If there exists the same amino acid category in one position, we can count 1. If there exists the same amino acid type, not just the same category, we can count 3. For three N-terminal motifs, the count values are 1, 3, and 4, respectively. For one C-terminal motif, the count value is 1. Then, we use a randomization experiment and iterate 1000 times, p-value for the N-terminal motifs is 0.032, and p-value for the C-terminal motif is 0.033. Consider the regular p-value as 0.05, the prediction results of our computational method is significant.

On all 16,000 peptide motifs, we identify the top 500 peptide motifs binding each 14-3-3 isoform. We identify six peptide motifs having 14-3-3*σ* specificity, as shown in Table 9. Compared with two 14-3-3*σ* preferable binders, two motifs have residues in the same amino acid category on *P*_−3_ and *P*_−1_ positions as shown in Table 1, on *P*_−3_ position, Ile along with Leu has Hydrophobic side chain; Pro and Gly are all special amino acids on *P*_+1_ position; and all of four C-terminal motifs show strong selection of Met and Tyr on *P*_+1_ and *P*_+2_ positions. As well as Leu and Val in same position of Lu’s motifs, they all have similar hydrophobic side chain.

**Table 9:** List of six preferable binders of 14-3-3*σ* from 16,000 peptide sequences.

No.	N-terminal	No.	C-terminal
1	HCDpS/TXXX	3	XXXpS/TMMG
2	ICPpS/TXXX	4	XXXpS/TMYH
		5	XXXpS/TYYC
		6	XXXpS/TYYK

## Discussion

We present a novel method for identifying peptide motifs binding to 14-3-3 isoforms. For each 14-3-3 isoform, we have 1,000 peptide motifs with experimental binding affinity values. We identify affinity values of 16,000 peptide sequences binding to seven 14-3-3 isoforms. First, we propose a sampling criteria to build a predictor for each new peptide motif. Then, we select nine physicochemical properties of amino acids and extract correlative properties of amino acids to describe each peptide motif. Finally, we consider elastic net to predict binding affinities of peptide motifs.

Our method tests 16,000 peptide motifs binding to seven distinct but highly homologous 14-3-3 isoforms, and the relative binding ability across six permutated positions similar with the experimental value. We identify phosphopeptides that preferentially bind to 14-3-3*σ* over other isoforms. Most of positions on peptide motifs are in the same amino acid category with experimental substrate specificity of phosphopeptides binding to 14-3-3*σ*. It indicates that, regardless of how the data are analyzed, 14-3-3*σ* consensus binding motifs derived from our experiments are in excellent agreement with previous work. Our method is designed and implemented as a generalized method that can be used to accurately predict the binding affinity for peptide-protein interaction in proteomics research.

## Supporting Information

S1 Table The cross-validation results over 1,000 peptides. (XLSX)
